# Can Disturbed Liver Perfusion Revealed in p-CT on the First Day of Acute Pancreatitis Provide Information about the Expected Severity of the Disease?

**DOI:** 10.1155/2019/6590729

**Published:** 2019-08-14

**Authors:** Joanna Pieńkowska, Katarzyna Gwoździewicz, Katarzyna Skrobisz, Monika Czarnowska-Cubała, Oliwia Kozak, Stanisław Hać, Michał Studniarek, Edyta Szurowska

**Affiliations:** ^1^II Department of Radiology, Faculty of Health Sciences, Medical University of Gdansk, Gdansk, Poland; ^2^I Department of Radiology, Faculty of Medicine, Medical University of Gdansk, Gdansk, Poland; ^3^Department of General Endocrine and Transplant Surgery, Medical University of Gdansk, Gdansk, Poland

## Abstract

**Background:**

The aim of the study was to evaluate the prognostic properties of perfusion parameters of liver parenchyma based on computed tomography (CT) of patients with acute pancreatitis (AP) made on the first day of onset of symptoms, to assess their usefulness in identifying patients with increased risk of the development of severe AP.

**Methods:**

79 patients with clinical symptoms and biochemical criteria indicative of AP underwent perfusion computed tomography (p-CT) within 24 hours after onset of the symptoms. Perfusion parameters in 41 people who developed a severe form of AP were compared with parameters in 38 patients in whom the course of AP was mild.

**Results:**

Statistical differences in the liver perfusion parameters between the group of patients with mild and severe AP were shown. The permeability-surface area product was significantly lower, and the hepatic arterial fraction was significantly higher in the group of patients with progression of AP.

**Conclusions:**

Based on the results, it seems that p-CT performed on the first day from the onset of AP is a method that, by revealing disturbances in hepatic perfusion, can help in identifying patients with increased risk of the development of severe AP.

## 1. Background

Acute pancreatitis (AP) is an inflammatory process, which, apart from the pancreas itself, can also involve the peripancreatic tissues or even other organs—if the inflammatory response is generalized [1–7].

In the majority of patients (approximately 75-80% of all cases), acute pancreatitis has a mild self-limiting course. The inflammatory reaction in such cases is local, and there is no multiple organ dysfunction syndrome (MODS) or concomitant systemic symptoms [3–5, 8–17]. However, in approximately 20-25% of patients, the course of AP can be severe; the mechanisms suppressing the local inflammatory reaction fail, which results in local tissue damage, pancreatic necrosis, infectious complications, and systemic inflammatory response syndrome (SIRS) [3, 18–24].

Local activation of inflammatory cells in AP triggers release of numerous mediators of systemic inflammatory response. They play a key role in the pathogenesis of the severe form of AP (SAP) and SAP-dependent MODS causing thromboembolic and ischemic complications leading to pancreatic necrosis [10]. Release of inflammatory mediators results in increased vascular permeability, which makes intravascular fluids enter the extravascular space, causing hypoperfusion and damage to many organs. Liver damage in acute pancreatitis confirmed histopathologically by experimental studies is also associated with abnormal perfusion during the systemic inflammatory process [10, 25–42]. Mortality rates in patients with organ failure in the course of SAP remain very high. It is estimated that the highest is associated with hepatic and renal failures (up to 83% and 63%, respectively) and the case of multiorgan failure is the highest for combined of hepatic and renal failures [18].

Perfusion CT is characterized by the high potential in the assessment of liver blood flow. It provides information on microcirculation in organ parenchyma and allows for the quantitative evaluation of the hemodynamic changes [43–49].

Since prognosis in liver damage remains poor, it seems very important to anticipate changes as early as possible. We believe that perfusion CT by detecting liver perfusion disorders could serve as a clinical helpful tool, enabling early diagnosis of the possible dysfunction of liver perfusion in the course of AP.

The aim of the study was to assess the prognostic properties of perfusion parameters of liver parenchyma, such as blood flow (BF), blood volume (BV), mean transit time (MTT), permeability-surface area product (PS), and hepatic arterial fraction (HAF). It was based on CT scans of patients with acute pancreatitis made on the first day of the onset of symptoms. The obtained perfusion values were analyzed to assess their usefulness in identifying patients with increased risk of the development of severe AP that can lead to multiorgan failure.

## 2. Material and Methods

The study protocol was approved by Independent Bioethics Commission for Research at the Medical University of Gdansk. All participants provided informed written consent for CT examination with intravenous contrast administration.

### 2.1. Patients

The final study material comprised of a group of patients aged 25–86 years (mean 47 years), including 32 women and 47 men with suspected acute pancreatitis who underwent p-CT scanning on the first day of onset of symptoms (Table 1). AP was diagnosed based on symptoms such as acute abdominal pain, nausea, vomiting, and increased levels of amylase and lipase in laboratory tests. On admission and on days 4-6 from AP onset, all patients had disease severity assessed with the use of the APACHE II scoring system. Laboratory parameters such as C-reactive protein (CRP), serum amylase and lipase levels, urine amylase levels, aspartate transaminase (AST), alanine transaminase (ALT), gamma-glutamyl transferase (GGT), alkaline phosphatase (ALP), and bilirubin levels were analyzed. The data were collected prospectively and then analyzed retrospectively.

Patients' recruitment criteria in this study were the same as in our previously published work on pancreatic perfusion [50]. All 41 patients, who after control CT carried out of the pancreas after 4-6 days from the onset of symptoms revealed progression of the disease, were included in the study. The control group was made up of the first 41 of all the patients in whom no progression of the disease was discovered. However, three of them had to be excluded from the study as in one of them, AP was related to pancreatic adenocarcinoma and in two others, exclusion was related to significant breathing/motion artifacts, which made it impossible to measure the perfusion in the optimal way. The remaining 38 patients without progression of AP were included in the final study.

The general exclusion criteria were as follows: history of allergy to iodine-containing contrast medium, renal failure, age below 18 years, pregnancy, and lack of consent to participate in the study.

### 2.2. CT Examinations

CT scans were performed on the first day of symptoms of acute pancreatitis (in accordance with the perfusion protocol) and on 4-6 days of the disease (standard method), using a 64-row GE CT scanner. In all patients, the first CT scanning involved assessment of the local perfusion with the measurement of the following parameters: BV, BF, MTT, PS, and HAF.

**Figure 1:**
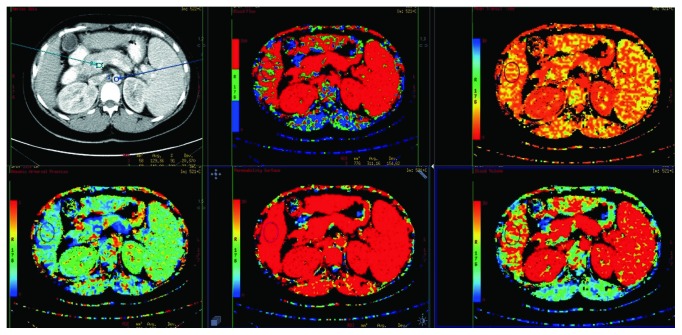
Point of reference located in the lumen of the abdominal aorta and portal vein and color perfusion maps for blood flow (BF), blood volume (BV), permeability-surface area product (PS), mean transit time (MTT), and hepatic arterial fraction (HAF).

In the liver, 4 oval or round fields were manually selected to form regions of interest (ROI), in which perfusion measurements were performed, avoiding large vessels and dilated bile ducts. Our point of reference was constituted by ROIs located manually in the lumen of the abdominal aorta and portal vein. The curve presenting arterial and portal enhancement and the colorful perfusion maps were generated automatically for each pixel representing a value of the analyzed parameter with the use of the dual-compartment Johnson-Wilson model (Figure 1).

The patients were divided into two groups according to the results of a follow-up CT scan: without progression of acute pancreatitis (patients with a mild form of pancreatitis—group I) and with progression of AP and occurrence of necrotic changes (subjects with severe acute pancreatitis—group II). Each of the groups was then divided into two subgroups. Patients with no progression were divided into a subgroup of subjects in whom none of the two scans (p-CT and control CT) revealed morphological changes (group IA) and another in whom mild pancreatic edema or the presence of free peripancreatic fluid was detected, which on the follow-up examination was shown to have completely regressed (group IB). Patients with progression of necrotic lesions were also divided into two subgroups: those with necrosis of the peripancreatic tissues only (group IIA) and those in whom necrosis involved pancreatic parenchyma or both the pancreatic parenchyma and the peripancreatic tissues (group IIB). We have divided the patients into subgroups based on the revised Atlanta classification [2], which distinguishes three subtypes of necrotizing pancreatitis depending on the anatomic area of necrotic involvement: pancreatic only, peripancreatic only, and combined pancreatic and peripancreatic.

### 2.3. Statistical Analysis

All statistical calculations were made with the use of StatSoft Inc. STATISTICA data analysis software system version 10.0 and Excel spreadsheet.

Quantitative variables were presented using the arithmetic mean, standard deviation, median, min. and max. values (range), and 95% CI (confidence interval). Qualitative variables were presented in numerical values and as percentages. For checking whether a quantitative variable came from a population of a standard distribution, Shapiro-Wilk *W* test was used. The significance of the differences between two groups was measured with the use of Student's *t*-test or Mann-Whitney *U* test. The significance of the differences between more than two groups was measured with the *F* test (ANOVA) or the Kruskal-Wallis test. Chi-square independence tests were applied for qualitative variables (Yates' correction for a cell count below 10, Cochran test, and Fisher's exact test).

## 3. Results

### 3.1. Patients

In a follow-up CT examination performed 4-6 days after onset of symptoms, 41 patients developed progression of AP with pancreatic and/or peripancreatic necrosis, while 38 other patients were diagnosed with a mild form of pancreatitis (group with no progression). Among 41 patients with progression of necrotic lesions, there were 19 in whom necrosis involved the peripancreatic tissues only (peripancreatic progression—group IIA) and 22 in whom it involved pancreatic parenchyma or both the pancreatic parenchyma and the peripancreatic tissues (pancreatic progression—group IIB). Thirty-eight patients with no progression of AP were divided into a subgroup of 25 subjects in whom none of the examinations revealed morphological changes (subgroup with no progression and no changes—IA) and a subgroup of 13 subjects in whom mild pancreatic edema and/or the presence of free peripancreatic fluid was detected, which on the follow-up examination showed to have completely regressed (subgroup with no progression and with changes—IB).

Transient organ dysfunction (respiratory or renal failure) developed in 4 patients. Significant complications in the form of infected necrosis occurred in 3 patients. There were no mortality cases in the study group.

In the assessed group, 74 people were admitted to hospital and their stay at the Department of Gastroenterology and Hepatology of the University Clinical Center in Gdansk, Poland, lasted between 5 and 24 days, on an average of 11 days. 4 of them were transferred to the intensive care unit. 5 patients with mild acute pancreatitis were released home after a short stay in the emergency ward (average 2 days).

Among patients with liver perfusion changes who developed a severe, necrotizing form of acute pancreatitis, 4 later required intervention in the form of endoscopic drainage of walled-off pancreatic necrosis.

Comparison of laboratory parameters (serum amylase and lipase levels, urine amylase levels, aspartate transaminase, alanine aminotransferase, gamma-glutamyl transferase, alkaline phosphatase, and bilirubin) and assessment of AP severity based on the APACHE II scoring system, conducted on both day 1 and then days 4-6, did not reveal statistically significant differences between the groups of patients with and without progression on a follow-up CT. Out of all laboratory tests performed on admission, only CRP values were significantly higher (*p* = 0.0001) in the group with progression, with a mean value of 113.17 mg/L (range 2.4-397.3), compared to 19.83 mg/L (range 0.6-227.2) in the group with no progression (Table 2).

### 3.2. Perfusion CT

In order to assess the effect of an inflammatory process on liver perfusion, 4 oval or round fields were manually selected and then comparative analysis was conducted between the groups with and without AP progression.

Manually selected ROIs in the liver were between 615.75 mm^2^ and 806.5 mm^2^. Since there were no statistically significant differences between perfusion values depending on the size of ROIs, they were presented as mean values (709.25 mm^2^).

In each of the four measurements of perfusion parameters in the liver, significantly lower PS values and significantly higher HAF values were observed in the whole group with progression, compared to the whole group of patients without progression. For this reason, the results are presented as mean values.

**Figure 2:**
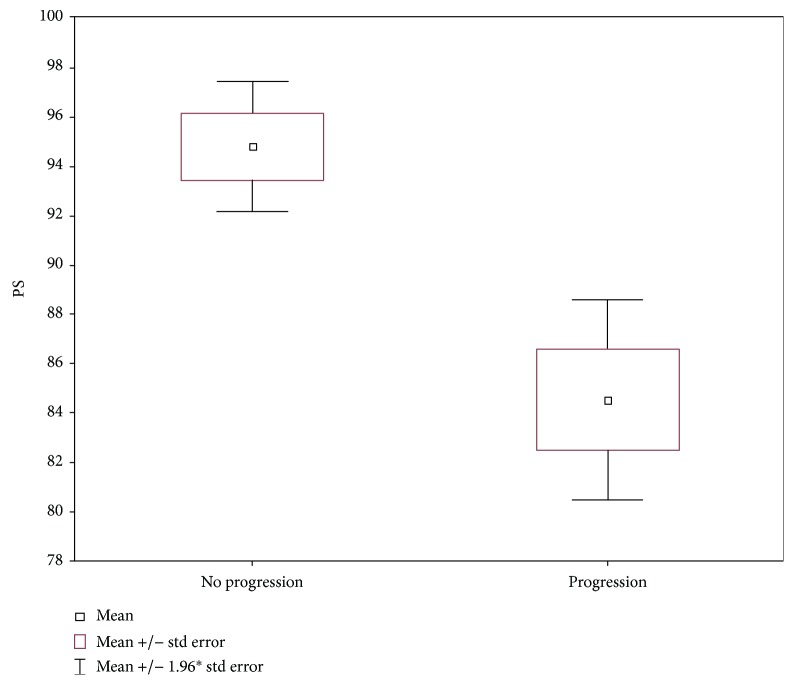
Mean PS values for the liver (groups I and II).

Mean values of PS for the liver were significantly lower (*p* = 0.0001) in the group of patients with progression compared to patients without progression and amounted to a mean value of 84.5 mL/100 g/min (*±*13.3) in patients with progression compared to a mean value of 94.8 mL/100 g/min (±8.2) in patients in whom there was no necrosis (Figure 2).

**Figure 3:**
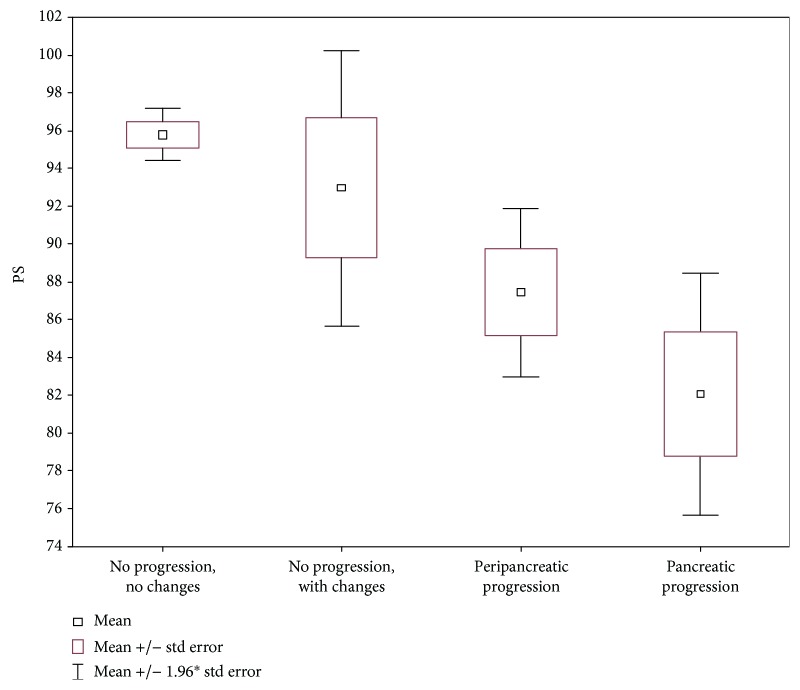
Mean PS for the liver (4 subgroups).

A comparison of four subgroups (IA, IB, IIA, and IIB) showed significantly higher values (*p* = 0.0001) in the subgroups without progression on a follow-up scan, compared to patients with progression and with the development of necrotic changes (Figure 3 and Table 3).

**Figure 4:**
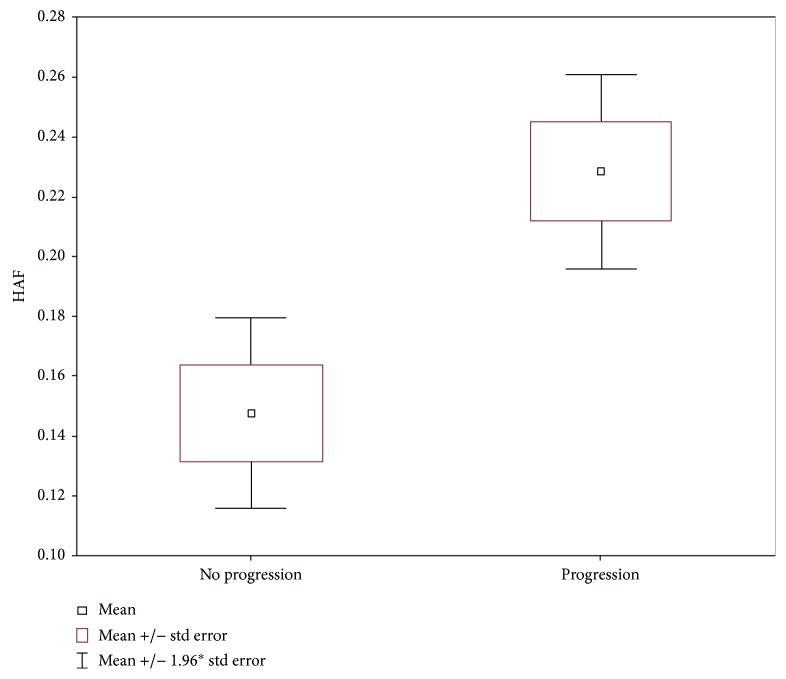
Mean HAF for the liver (groups I and II).

Comparative analysis of HAF between groups with and without progression in the course of AP showed that HAF was significantly higher in the group of patients with necrotic progression, compared to patients without progression (*p* = 0.0004). Mean HAF values were 0.23 (±0.11) in the group with progression (group II), compared to 0.15 (±0.10) in the group without progression (group I) (Figure 4).

**Figure 5:**
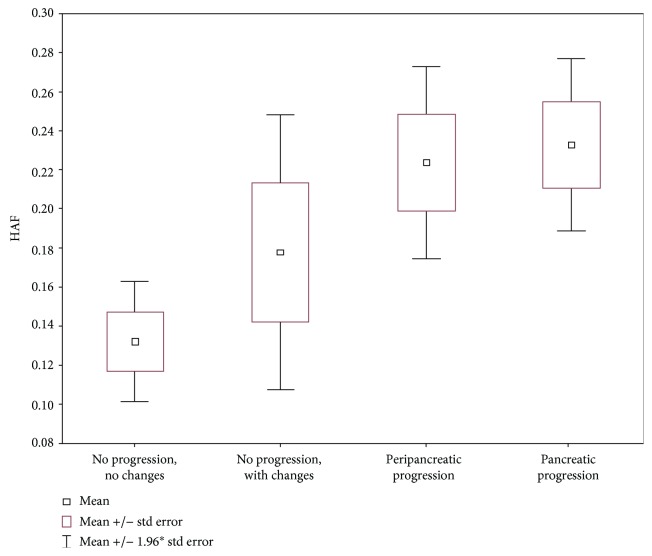
Mean HAF for the liver (4 subgroups).

Based on a comparison of the four subgroups, it was observed that HAF was also significantly higher in both subgroups of patients with progression of the disease (*p* = 0.0040) (Table 4 and Figure 5).

There were no statistically significant differences observed between the values of other measured perfusion parameters for the liver: BF (*p* = 0.1246), BV (*p* = 0.0853), and MTT (*p* = 0.9335) neither between the main two groups nor between all four subgroups.

## 4. Discussion

Despite diagnostic and therapeutic progress, the mortality rate for severe acute pancreatitis remains high and amounts to as much as 15–25% [19, 29, 51–53]. In most cases, it is associated with multiorgan failure which occurs in as many as 70% of patients with SAP. The number of deaths in this group of patients is almost 10 times higher compared to patients without multiple organ dysfunction syndrome [4, 18, 19, 27–29, 54]. Due to the high mortality rate of patients with severe AP, attempts have been made to identify patients in whom multiple organ dysfunction syndrome can be predicted in order to intensify therapeutic management.

Various prognostic scales are used to predict the severity of the course of AP, the development of organ failure, and the appearance of pancreatic necrosis. Most clinical scoring systems are calculated 24 to 48 hours after disease onset. APACHE II allows prediction of organ failure after 24 hours and Ranson's scale after 48 hours. It seems that for predicting organ failure, CT perfusion has lower efficacy compared to the clinical scoring system. On the other hand, p-CT allows early prediction of a severe form of AP which predisposes to infected necrosis and organ failure, both of which are the main factors determining mortality. Considering the limited value of the clinical scales, it is worth taking into account the precise method which is p-CT as it may provide important information on the probability of developing necrotizing pancreatitis earlier than 24-48 hours and thus indirectly indicate a threat of multiorgan failure. Of course, it might be questioned whether this prediction method would change clinical management. It seems, however, that the evidence of early microcirculation changes in AP can be translated into clinical decisions and may be important, for example, to allow management resolutions that may include aggressive hydration, anticoagulation therapy, or referral to an intensive care unit.

The study material comprised a group of 79 patients diagnosed with AP made on the basis of the overall clinical picture and laboratory tests. In our material, all patients underwent p-CT scan on the first day of the onset of symptoms of acute pancreatitis. We assessed the parameters of hepatic perfusion and then compared the obtained results between groups of patients with and without AP progression (mild or severe form of pancreatitis), diagnosed on the basis of a follow-up CT scan performed 4-6 days later.

The increased risk of liver dysfunction in the course of acute pancreatitis was confirmed in experiment conducted on rats which had AP induced by intraperitoneal administration of caerulein. At the end of the experiment, the rats were sacrificed; their livers were excised, and the hepatic tissue was sent for histopathological analysis, which showed hepatocyte destruction, sinusoidal dilatation, focal necrosis, Kupffer cell proliferation, and central vein congestion [34].

Despite the proven effect of AP on the liver, the problem of liver perfusion disturbances is rarely raised. One of the very few reports pertaining exclusively to this issue, presented by Koyasu et al., described 67 patients with severe AP who underwent p-CT scanning within 72 hours from the onset of the first symptoms [35]. Also, Tsuji et al. and Yadav et al., who assessed not only pancreatic perfusion but also liver perfusion, conducted the examinations during the first three days following the onset of AP symptoms [51, 52].

In our study, we have correlated the values of the parameters indicating liver perfusion disturbances with the severity of acute pancreatitis, due to the fact that the severe form of AP predisposes to infected necrosis and organ failure, both of which are the main factors determining mortality.

As it is well known, in the course of acute pancreatitis, a renal failure and respiratory failure are the most common. Hepatic failure is rare, which is associated with its large functional reserve. Based on this, it seems that the degree of liver perfusion disorders, which, as we have shown, is greater in cases of a severe form of pancreatitis, may indicate the possibility of multiorgan failure development.

The volume of hepatic blood is estimated to be at 1/4 of the cardiac output and is significant for maintaining normal liver function. Decrease in perfusion values in the liver impairs its functions by suppressing the blood/hepatocyte replacement process. In the presented material, 4 oval or round fields (ROIs) were manually selected in the liver and perfusion measurements were performed there. The dual-compartment Johnson-Wilson model was applied for the assessment of perfusion, and statistically significant differences in the PS and HAF values were observed for the group of patients without progression of acute pancreatitis vs patients with progression. However, there were no statistically significant differences observed for the values of all other investigated perfusion parameters (BF, BV, and MTT).

In our study, mean values of the permeability-surface area product for the contrast medium passing from the intravascular to the extravascular space for the liver were significantly lower (*p* = 0.0001) in the group and both subgroups of patients with progression compared to the patients without progression. Since the permeability-surface area product reflects the rate at which contrast medium passes from the capillary endothelium to the intercellular space, it seems that the decrease in the values of this parameter in patients with AP progression can result from the disturbances in the organ's function and impaired blood/hepatocyte replacement process. At the later stages of the disease, the decrease in PS values can be associated with the decreased stroke volume in patients with MODS and decreased volume of hepatic blood. Due to the fact that there are no study reports available referring to this issue, the results obtained by us should be confirmed in other experiments.

Hepatic arterial fraction represents the share of blood entering the liver through the hepatic artery in relation to the total amount of blood entering it. In this article, comparative analysis of HAF between both groups and four subgroups showed that HAF was significantly higher in the whole group and both subgroups of patients with necrotic progression (*p* = 0.0004) and that HAF values increased with progression of the inflammatory process. In a normally functioning liver, approximately 3/4 of the blood supply comes from the portal vein and only about 1/4 is from the hepatic artery. Higher HAF values in patients with AP progression indicate higher blood supply to the liver through the hepatic artery, which was also observed in studies by other authors. It was concluded that the inflammatory process can increase the arterial supply and decrease the venous one, not only in the course of AP but also in cholecystitis, hepatic abscess, or cholangitis [34, 55]. In the study by Koyasu et al., the authors observed that patients with SAP had significantly higher arterial blood flow compared to the control group with the healthy pancreas [35]. The results obtained by us confirmed the increased supply in arterial blood in the liver as early as on day one of AP and showed significant differences between the volume of arterial inflow in relation to the further course of the disease—the values were the highest in patients who later developed pancreatic necrosis. Due to the connection between SAP and organ failure, it seems that this parameter can prove useful in the prediction of the further course of the disease.

In the presented material, there were no statistically significant differences observed between the values of other measured perfusion parameters for the liver—BF (*p* = 0.1246), BV (*p* = 0.0853), and MTT (*p* = 0.9335), neither between the main two groups nor between all four subgroups. Several authors of available reports observed that BF and BV values differ between groups of patients with and without AP progression. However, it should be remembered that all these examinations were conducted within up to 72 hours and not up to 24 hours from the onset of clinical symptoms as in the case of our study, which can explain the differences in the obtained results. Another explanation for why we did not observe any statistically significant differences may be the possibility that the increase of the arterial supply in the liver was compensated by a decrease in the venous supply.

The current clinical guidelines state that early CT imaging is not required in all patients with acute pancreatitis. The main reason for the recommendation against the routine use of the early cross-sectional imaging is the fact that pancreatic necrosis develops in the course of severe AP usually within 72 hours of the onset of the disease. Therefore, in the first 24-48 hours of the disease, the CT images may be ambiguous. Hence, according to the current guidelines, if the patient meets the criteria for acute pancreatitis on the basis of symptoms and laboratory tests, early CT is not required to confirm the diagnosis.

There are, however, publications that indicate the benefit of an early CT imaging, but not by the standard method but by using the CT perfusion option [56, 57]. CT perfusion allows the detection and quantification of early microvascular changes in tissues and may be a promising technique for predicting tissue viability. As we have shown in our previous work [50], the extent of microcirculation derangements correlates well with the disease severity and with the necrotic areas on follow-up computed tomography. Perfusion CT is therefore a method that has the potential to select a group of patients at an earlier stage of the disease who are at risk of developing a severe form of AP in the course of which organ failure occurs more frequently. Considering the constant development of imaging techniques and the use of low-dose CT scans, which are beneficial to patients due to the reduction of their exposure to radiation, perhaps, perfusion CT is a method that over time will be used in the early diagnosis of acute pancreatitis and will be included in the guidelines.

The results presented in our report must obviously be confirmed in other examinations performed within the first 24 hours from the onset of the clinical symptoms of AP, along with assessment of all other perfusion parameters that we measured.

The disadvantages of the study include the fact that during the observation, based on the laboratory tests performed after 4-6 days following the onset of the disease, in the group of evaluated patients, there were no direct symptoms of multiple organ failure but only progression of AP in some of them. Nevertheless, patients with AP progression are threatened with MOF complications. We believe that the changes that we noticed may also be the early markers of reversible disorders, which may either completely withdraw or lead to a multiple organ failure unless modification of the treatment, influenced by p-CT images, is applied.

## 5. Conclusion

To conclude, based on the obtained results, it seems that p-CT performed within the first 24 hours from the onset of AP is a method which, by revealing disturbances in hepatic perfusion, may predict the possible evolution of pancreatitis to SAP and MOF and allow to isolate groups of patients in whom we can expect an unfavorable course of the disease.

## Figures and Tables

**Table 1:** Characteristics of enrolled patients.

Total number of enrolled patients	79
Age (years)	47, 2 ± 16, 0
Gender (male/female)	47/32
Etiology (%)	
Alcoholic	38 (48,1)
Biliary	23 (29,1)
Idiopathic	4 (5,1)
Acute on chronic	8 (10,1)
ERCP related	4 (5,1)
Hypertriglyceridemia	2 (2,5)
APACHE II (group I)	1-8, mean 3,6
APACHE II (group II)	1-8, mean 4,1

**Table 2:** Laboratory test value of enrolled patients.

	Group I (*n* = 38)	Group II (*n* = 41)	Total (*n* = 79)	*p*
*CRP*				
Mean (SD)	19,83 (37,52)	113,17 (98,37)	68,27 (88,51)	
95% CI	(7,50; 32,16)	(82,12; 144,22)	(48,45; 88,10)	
Range (min-max)	0,60-227,20	2,40-397,30	0,60-397,30	
Median	8,25	112,50	18,70	0,0001
*Serum lipase*				
Mean (SD)	2685,55 (3136,28)	1358,34 (1807,69)	1996,75 (2605,19)	
95% CI	(1654,68; 3716,42)	(787,77; 1928,92)	(1413,22; 2580,28)	
Range (min-max)	38,00-12596,00	26,00-7197,00	26,00-12596,00	
Median	1344,00	568,00	720,00	0,0609
*Serum amylase*				
Mean (SD)	1092,26 (970,36)	693,07 (768,50)	885,09 (888,71)	
95% CI	(773,31; 1411,21)	(450,50; 935,64)	(686,03; 1084,15)	
Range (min-max)	107,00-3956,00	27,00-3429,00	27,00-3956,00	
Median	678,50	295,00	571,00	0,0095
*Urine amylase*				
Mean (SD)	5883,68 (5828,18)	6324,71 (10772,69)	6112,57 (8699,16)	
95% CI	(3968,01; 7799,36)	(2924,43; 9724,99)	(4164,06; 8061,07)	
Range (min-max)	265,00-18675,00	220,00-41395,00	220,00-41395,00	
Median	3142,00	2155,00	2778,00	0,1187
*AST*				
Mean (SD)	168,68 (183,72)	124,39 (139,79)	145,70 (162,87)	
95% CI	(108,30; 229,07)	(80,27; 168,51)	(109,21; 182,18)	
Range (min-max)	15,00-839,00	5,00-581,00	5,00-839,00	
Median	93,50	57,00	76,00	0,1130
*GGT*				
Mean (SD)	326,32 (279,93)	398,29 (450,41)	363,67 (377,51)	
95% CI	(234,30; 418,33)	(256,13; 540,46)	(279,11; 448,23)	
Range (min-max)	17,00-867,00	10,00-2055,00	10,00-2055,00	
Median	285,00	203,00	235,00	0,5300
*ALP*				
Mean (SD)	209,61 (282,13)	114,56 (70,30)	160,28 (206,34)	
95% CI	(116,87; 302,34)	(92,37; 136,75)	(114,06; 206,50)	
Range (min-max)	36,00-1445,00	37,00-306,00	36,00-1445,00	
Median	124,00	79,00	102,00	0,0583
*Bilirubin*				
Mean (SD)	2,89 (2,81)	2,90 (3,36)	2,90 (3,09)	
95% CI	(1,97; 3,82)	(1,84; 3,96)	(2,21; 3,59)	
Range (min-max)	0,23-10,97	0,42-13,75	0,23-13,75	
Median	1,90	1,11	1,37	0,8830

CRP: C-reactive protein; AST: aspartate transaminase; ALT: alanine transaminase; GGT: gamma-glutamyl transpeptidase; ALP: alkaline phosphatase; group I: mild form of acute pancreatitis; group II: severe form of acute pancreatitis.

**Table 3:** Comparison of 4 subgroups with regard to mean PS.

Liver PS	No progression with no changes	No progression with changes	Peripancreatic progression	Pancreatic progression	*p*
Mean (SD)	95.8 (3.5)	93.0 (13.4)	87.4 (10.0)	82.0 (15.4)	*p* = 0.0001
95% CI	(94.3; 97.2)	(84.9; 101.0)	(82.6; 92.2)	(75.2; 88.9)	
Range (min-max)	86.6-101.1	49.9-100.6	54.3-96.0	46.1-98.6	
Median	95.1^1,2^	98.2^3,4^	90.9^1,3^	84.7^2,4^	

**Table 4:** Comparison of 4 subgroups with regard to mean HAF.

Liver HAF	No progression with no changes	No progression with changes	Peripancreatic progression	Pancreatic progression	*p*
Mean (SD)	0.13 (0.08)	0.18 (0.13)	0.22 (0.11)	0.23 (0.11)	*p* = 0.0040
95% CI	(0.10; 0.16)	(0.10; 0.26)	(0.17; 0.28)	(0.19; 0.28)	
Range (min-max)	0.03-0.26	0.06-0.38	0.09-0.46	0.13-0.42	
Median	0.12^1,2^	0.10	0.19^1^	0.20^2^	

## Data Availability

The imaging and statistical data used to support the findings of this study may be released upon application to Joanna Pienkowska (jpienkowska@gumed.edu.pl) of the Department of Radiology, Medical University of Gdansk, Poland, who can be contacted at the 2nd Department of Radiology, Medical University of Gdansk, Smoluchowskiego 17, 80-952 Gdansk, Poland.

## Abbreviations

CT: Computed tomography
AP: Acute pancreatitis
p-CT: Perfusion computed tomography
MODS: Multiple organ dysfunction syndrome
SIRS: Systemic inflammatory response syndrome
SAP: Severe acute pancreatitis
BF: Blood flow
BV: Blood volume
MTT: Mean transit time
PS: Permeability-surface area product
HAP: Hepatic arterial fraction
ROI: Regions of interest.
